# Protein tyrosine phosphatase 1B is a mediator of cyclic ADP ribose-induced Ca^2+^ signaling in ventricular myocytes

**DOI:** 10.1038/emm.2017.68

**Published:** 2017-06-02

**Authors:** Seon-Ah Park, Bing-Zhe Hong, Ki-Chan Ha, Uh-Hyun Kim, Myung-Kwan Han, Yong-Geun Kwak

**Affiliations:** 1 Department of Pharmacology, Jeonju, Republic of Korea; 2 Department of Biochemistry, Jeonju, Republic of Korea; 3 Department of Microbiology, Institute for Medical Sciences, Chonbuk National University Medical School, Jeonju, Republic of Korea

## Abstract

Cyclic ADP-ribose (cADPR) releases Ca^2+^ from ryanodine receptor (RyR)-sensitive calcium pools in various cell types. In cardiac myocytes, the physiological levels of cADPR transiently increase the amplitude and frequency of Ca^2+^ (that is, a rapid increase and decrease of calcium within one second) during the cardiac action potential. In this study, we demonstrated that cADPR levels higher than physiological levels induce a slow and gradual increase in the resting intracellular Ca^2+^ ([Ca^2+^]_i_) level over 10 min by inhibiting the sarcoendoplasmic reticulum Ca^2+^ ATPase (SERCA). Higher cADPR levels mediate the tyrosine-dephosphorylation of α-actin by protein tyrosine phosphatase 1B (PTP1B) present in the endoplasmic reticulum. The tyrosine dephosphorylation of α-actin dissociates phospholamban, the key regulator of SERCA, from α-actin and results in SERCA inhibition. The disruption of the integrity of α-actin by cytochalasin B and the inhibition of α-actin tyrosine dephosphorylation by a PTP1B inhibitor block cADPR-mediated Ca^2+^ increase. Our results suggest that levels of cADPR that are relatively higher than normal physiological levels modify calcium homeostasis through the dephosphorylation of α-actin by PTB1B and the subsequent inhibition of SERCA in cardiac myocytes.

## Introduction

Ca^2+^ plays a fundamental role in the cardiac contraction and relaxation cycle by linking the electrical depolarization of cardiomyocytes with contraction (that is, excitation–contraction coupling; EC coupling).^1^ Cellular depolarization after the action potential is generated from the sinoatrial node activates voltage-operated Ca^2+^ channels, which causes an influx of Ca^2+^ across the sarcolemma and into the cytoplasm.^1^ The resulting Ca^2+^ influx activates ryanodine receptors (RyRs) on the sarcoplasmic reticulum (SR), which causes more Ca^2+^ to be released into the cytosol—this phenomenon is known as Ca^2+^-induced Ca^2+^ release (CICR).^2, 3, 4^ A transient increase in the free cytosolic calcium concentration ([Ca^2+^]_i_), Ca^2+^ transient in cardiac myocytes allows the actin and myosin contractile filaments to engage and slide past each other, resulting in cardiac muscle contraction.^4^ Sarcoendoplasmic reticulum Ca^2+^ ATPase (SERCA) in sarcoplasmic reticulum (SR) in cardiac myocytes transfers Ca^2+^ from the cytosol to the lumen of the SR as a result of ATP hydrolysis during muscle relaxation.^5^ The transfer of Ca^2+^ by SERCA from the cytosol to the SR is inhibited by unphosphorylated phospholamban (PLB).^6^ PLB phosphorylation can relieve the inhibition of the SERCA pump and enhance [Ca^2+^]_i_.^6^

Cyclic ADP-ribose (cADPR) is synthesized from NAD+ by bifunctional ectoenzymes including CD38 and CD157, and monofunctional ADP ribosyl cyclase from the Aplysia mollusc.^7, 8, 9^ In cardiac myocytes, nanomolar cADPR concentrations increase the amplitude and frequency of Ca^2+^ transient through an increased accumulation of Ca^2+^ in the SR and the subsequent luminal Ca^2+^-dependent activation of RyRs.^10^ In this study, we investigated the mechanism by which micromolar cADPR concentrations affect [Ca^2+^]_i_ in cardiac myocytes.

## Materials and methods

### Materials

The reagents 3-(3,5-dibromo-4-hydroxy-benzoyl)-2-ethyl-benzofuran-6-sulfonic acid-(4-(thiazol-2-ylsulfamyl)-phenyl)-amide a PTP inhibitor; 8-hydroxy-7-(6-sulfonaphthalen-2-yl)diazenyl-quinoline-5-sulfonic acid, an SHP1/2 PTPase inhibitor; and sodium stibogluconate were from Calbiochem (San Diego, CA, USA). Fura 2 AM and Fura 2 lowaff were obtained from Invitrogen (Carlsbad, CA, USA) and TEFLabs (Austin, TX, USA), respectively.

### Preparation of cardiac myocytes

This study was approved by the institutional review committee of Chonbuk National University (Reference Number: CBU 2008-0057). New Zealand white rabbits (1.8–2.4 kg) were anesthetized with an intramuscular injection of 50 mg kg^−1^ ketamine and 20 mg kg^−1^ xylazine hydrochloride. The hearts were removed after deep anesthesia was confirmed by the disappearance of the corneal reflex and the withdrawal of the hindlimb resulting from clamping of the paw. Cardiac myocytes were enzymatically isolated from the ventricle as previously described^11^ and superfused at 34−36 °C with a solution containing (mM) 8.5 NaCl, 14.5 NaHCO_3_, 4.2 KCl, 1.18 MgSO_4_·7H_2_O, 2.5 CaCl_2_ and 11.1 glucose (oxygenated at 95% O_2_, 5% CO_2_).

### Fluorimetric determination of [Ca^2+^]_i_

Rabbit ventricular myocytes were loaded with fura 2-AM (5 μM) through a 60-min incubation. After they were washed, the cells were seeded in a 200-μl well with a glass coverslip on the bottom and incubated on the stage of an inverted fluorescence microscope (Nikon, Tokyo, Japan) that was continuously perfused at 37 °C. The fluorescence was measured at a determined site through a pinhole with alternating excitation wavelengths of 340 and 380 nm and an emission wavelength of 510 nm using a Ca^2+^ microspectrofluorometer (PTI). At the end of each recording, the data were calibrated in terms of [Ca^2+^]_i_ as described by Grynkyewicz *et al.* based on equation (a)^12^ and normalized to calculate the % increase of [Ca^2+^]_i_ (b)^6, 13, 14, 15^ A *K*_d_ value of 229 nM was assumed for the binding of Ca^2+^ to fura 2-AM. R_max_, R_min_, Sf2 and Sb2 were measured in each experimental cell by the addition of 20 mM CaCl_2_ (R_max_) and 50 mM EGTA (R_min_).


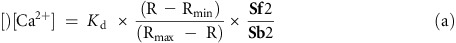



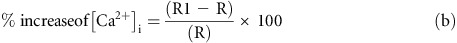


At the beginning of each experiment, the cells were washed in a Ca^2+^-free solution containing (mM) 135 NaCl, 5.4 KCl, 1 MgCl_2_, 10 glucose, and 5 HEPES. Patch-pipettes were pulled from borosilicate glass capillary tubules by a micropipette puller (PP-83, Narishige Co. Ltd, Tokyo, Japan) and fire-polished. The patch-pipette tip resistance was between 4–6 MΩ. The intracellular solution for filling each patch pipette contained 110 mM KCl, 5 mM K_2_ATP, 10 mM HEPES (pH 7.2 with KOH). After attachment to the myocytes, the resistance was monitored beginning when the giga seal formation (Axon AxoScope 10). cADPR (100 μM) or IP_3_ (100 nM) was applied to the cytosol by rupturing the plasma membrane with a patch-pipette containing each reagent. The reagents 8-Bromo cADPR (Br-cADPR), ryanodine, caffeine, dantrolene, xestospongin, tetracaine, the PTP1B inhibitor, the SHP1/2 PTPase inhibitor or sodium stibogluconate were applied to the bath. Thapsigargin was also applied to the cytosol with a patch pipette.

### Immunoprecipitation and immunoblotting

The isolated ventricular myocytes were lysed in lysis buffer containing (in mM) 150 NaCl, 20 HEPES, 1 EDTA, and 0.1 PMSF, as well as 1% Triton X-100 (in μg ml^−1^), at pH 7.2, and the lysates were centrifuged at 13,000 *g* for 10 min. The proteins were then immunoprecipitated with rabbit IgG (Sigma), mouse anti-SERCA (Affinity BioReagents), mouse anti-α-actin (Sigma) or mouse anti-PLB antibodies (Affinity BioReagents) (1:100 dilution). The immune complexes were subsequently collected by adding protein A or G beads (1/10 volume, Sigma), fractionated by 10% SDS-PAGE, and transferred to polyvinylidene difluoride membranes. The blots were incubated with anti-phosphotyrosine (Santa Cruz Biotechnology, Inc., Dallas, TX, USA), anti-α-actin, anti-PLB antibodies, anti-phospho-Ser,^16^ or anti-phospho-Thr^17^ PLB antibodies (Badrilla, Leeds, UK) and subsequently incubated with goat anti-mouse or anti-rabbit alkaline phosphatase-conjugated secondary antibodies (Santa Cruz Biotechnology). The proteins were visualized using an enhanced chemiluminescence system (Intron, Seongnam, Republic of Korea) and an LAS 3000 imaging system (Fuji, Tokyo, Japan). To detect the total or tyrosine-phosphorylated α-actin in SR vesicles, we removed the IgG heavy chain band using ImmunoCruzTM IP/WB Optima E (Santa Cruz Biotechnology, Inc.) according to the manufacturer’s instructions.

### Fluorimetric measurement of SR Ca^2+^ uptake

Rabbit SR vesicles (0.5 mg per 1 ml cuvette) were pre-incubated for 1 min at room temperature in the SR Ca^2+^ uptake solution containing (mM) 50 KCl, 20 MOPS, 0.01 CaCl_2_, 5 NaN_3_, 1 KH_2_PO_4_, 5 creatine phosphate, and 10 U creatine phosphokinase. Rabbit SR vesicles (0.5 mg per 1 ml cuvette) suspended in a cuvette were mixed with 1 μM Fura-2 lowAff. The Fura 2 fluorescence outside of the vesicles was monitored over time with alternating excitation wavelengths of 340 and 380 nm and an emission wavelength of 510 nm, using a Ca^2+^ microspectrofluorometer (PTI). The extravesicular Ca^2+^ concentration was expressed as a fluorescence excitation ratio (*R*_340nm/380nm_).

### Identification of proteins

The dried samples were analyzed by matrix-assisted laser desorption/ionization-time-of flight (MALDI-TOF) mass spectrometry (Voyager-DE PRO) for peptide mass fingerprinting and by electrospray ionization quadrupole time of flight (ESI-Q-TOF) mass spectrometry for peptide sequencing. Database searches were carried out using MS-Fit, accessed via the World Wide Web at http://prospector.uscf.edu.

### Measurement of ATPase activity in intact SR vesicles

The ATPase activity in the SR vesicles was determined using an ATPase assay kit (Innova Biosciences, Cambridge, UK) according to the manufacturer’s instructions with minor modifications. Briefly, SR vesicles (0.1 μg) in a total volume of 100 μl were incubated for 10 min in the presence of various concentrations of cADPR (0–40 μM) with or without a 10 min pretreatment with various inhibitors. The mixture was incubated for 1 min at 37 °C in substrate buffer (0.5 M Tris-HCl, 0.1 M MgCl_2_, 10 mM purified ATP, 5 μM calcein-AM, 5 mM sodium azide, and 1 mM ouabain, pH 7.4) with or without Na-orthovanadate (2–20 μM). After incubation, the reaction was stopped by the addition of 50 μl of Gold Mix from the ATPase assay kit. Two minutes later, 20 μl of stabilizer from the ATPase assay kit was added, and the solution was incubated for 20 min at 37 °C in the dark. The enzyme activity was calculated by measuring the Pi-dye complex released via ATP hydrolysis using an ELISA plate reader at 635 nm (Molecular Devices, Sunnyvale, CA, USA).

### Statistical analysis

Values are expressed as the mean±s.e.m. Significant differences were determined by Student’s *t-*test; *P*<0.05 was considered significant.

## Results

### cADPR induces an increase in the resting intracellular Ca^2+^ concentration ([Ca^2+^]_i_) in isolated rabbit ventricular myocytes independently of ryanodine- and IP_3_-sensitive pools

**Figure 1:**
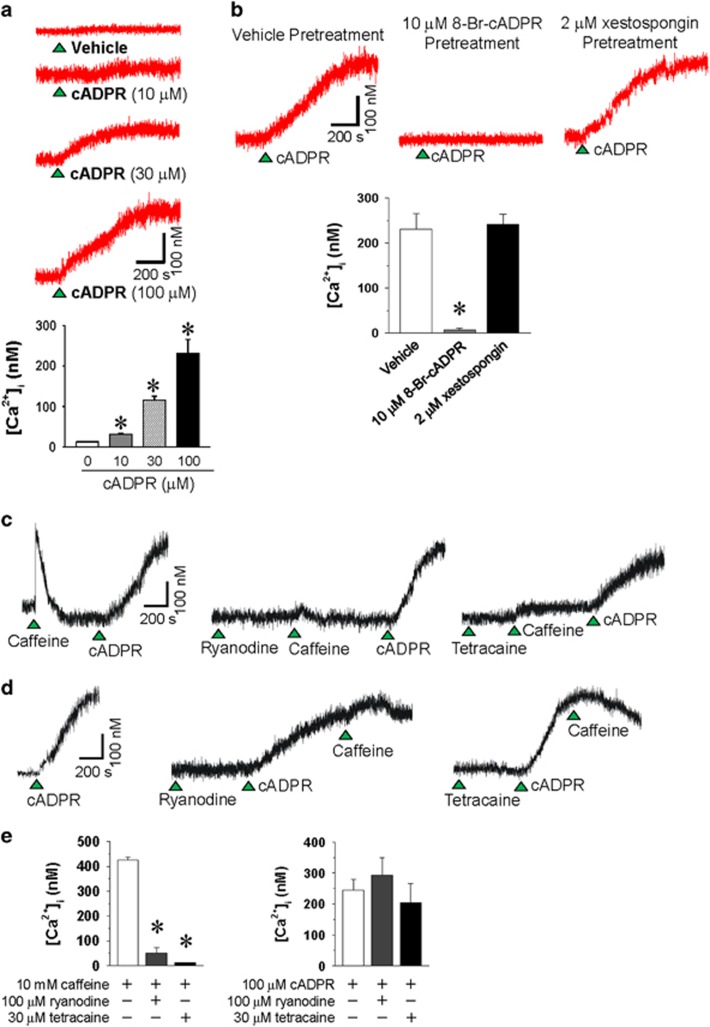
cADPR induces an increase in resting [Ca^2+^]_i_ in isolated rabbit ventricular myocytes independent of ryanodine- and IP_3_-sensitive pools. (**a**) The effect of intracellular cADPR application on resting [Ca^2+^]_i_ in isolated rabbit ventricular myocytes. cADPR was dialyzed through a patch pipette into rabbit ventricular myocytes loaded with Fura 2-AM. [Ca^2+^]_i_ was measured with a Ca^2+^ microspectrofluorometer equipped with an inverted fluorescence microscope as described in *Materials and Methods*. The arrowheads indicate the starting point of cADPR perfusion or the rupturing of the cell membrane attached to the patch pipette. The tracings represent six experiments. Columns with vertical bars represent the mean±s.e.m. of [Ca^2+^]_i_ from six experiments. *P<0.05 vs the basal level of [Ca^2+^]_i_. (**b**) The effect of 8-Br-cADPR and xestospongin pretreatment on cADPR-induced increase in resting [Ca^2+^]_i_ in isolated rabbit ventricular myocytes. Fura 2-AM–loaded cells were incubated for 20 min with 10 μM 8-Br-cADPR, and 2 μM xestospongin before perfusion with 100 μM cADPR. The tracings represent six experiments. Columns with vertical bars represent the mean±s.e.m. of [Ca^2+^]_i_ from six experiments. *P<0.05 vs vehicle pretreatment. (**c**, **d**) The effect of ryanodine (**c**) or tetracaine (**d**) on cADPR- or caffeine-induced [Ca^2+^]_i_. Ryanodine (100 μM) or tetracaine (30 μM) was applied to the bath and incubated for 5 min. Caffeine (10 mM) was applied and then 100 μM cADPR was perfused into the cell through a patch pipette (**c**). Inversely, intracellular 100 μM cADPR was perfused into the cell through a patch pipette and then 10 mM caffeine was applied to the bath (**d**). The tracings represent three experiments. (**e**) The effect of ryanodine and tetracaine on the caffeine- (right panel) and cADPR- (left panel) induced Ca^2+^ increase. Columns with vertical bars represent the mean±s.e.m. of [Ca^2+^]_i_ from three experiments. *P<0.05 vs caffeine-induced [Ca^2+^]_i_ increase with vehicle pretreatment.

To investigate the effect of higher cADPR levels on resting [Ca^2+^]_i,_ we intracellularly applied cADPR at micromolar levels in the cytosol through a patch pipette in rabbit ventricular myocytes and monitored resting [Ca^2+^]_i._ Intracellular cADPR application increased resting [Ca^2+^]_i_ slowly in a concentration-dependent manner, reaching a maximum at 100 μM cADPR (Figure 1a). When the cells were pretreated with 10 μM 8-Br-cADPR (a competitive cADPR antagonist) for 10 min, the intracellular cADPR application had no effect on the resting [Ca^2+^]_i_ (Figure 1b), indicating that cell dialysis by patch pipette itself did not change the [Ca^2+^]_i_. Pretreatment with xestospongin C (2 μM), an IP_3_ antagonist, did not affect cADPR-induced increase in resting [Ca^2+^]_i_ (Figure 1b). Pretreatment with ryanodine, a RyR blocker, and tetracaine, a RyR2 inhibitor, blocked caffeine-induced but not cADPR-induced increase in resting [Ca^2+^]_i_ (Figures 1c and e). These results indicate that cADPR-induced an increase in resting [Ca^2+^] in cardiac myocytes that did not involve RyRs and IP_3_ receptors.

**Figure 2:**
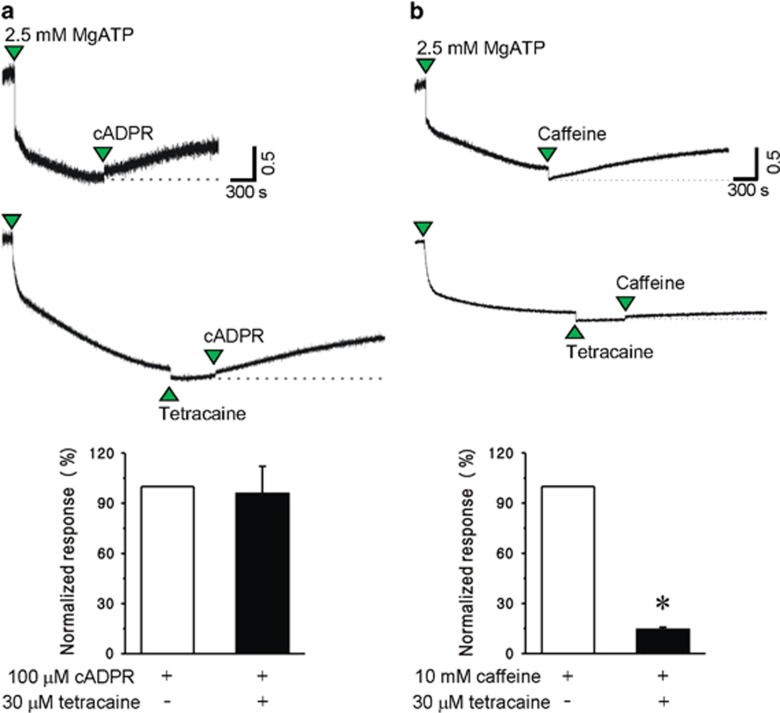
Effect of tetracaine on cADPR- and caffeine-induced Ca2+ release from isolated SR vesicles. (**a**, **b**) Rabbit SR vesicles (0.5 mg per 1 ml cuvette) suspended in a cuvette were mixed with 1 μM Fura-2 lowAff. The Fura 2 fluorescence outside of the vesicles was monitored with time. MgATP (2.5 mM) was added to the mixture to take up Ca^2+^ into the SR. After the uptake reaction achieved a steady state, 100 μM cADPR (**a**) or 10 mM caffeine (**b**) was applied in the absence (upper panels) or presence (lower panels) of 10 μM tetracaine. The tracings represent three experiments. Columns with vertical bars represent a quantitative summary of maximum 340/380 nm excitation ratios for Fura-2 LowAff after cADPR or caffeine addition.

To prevent the perturbation of Ca^2+^ signaling by Fura 2-mediated Ca^2+^ buffering and the effect of Ca^2+^ influx by calcium channels in the plasma membrane, we investigated whether cADPR induces a Ca^2+^ release from isolated SR vesicles using Fura-2 lowAff, a low affinity calcium chelator. The addition of ATP rapidly decreased extravesicular [Ca^2+^] (Figures 2a and b) due to Ca^2+^ chelation followed by Ca^2+^ uptake into SR vesicles via an SR Ca^2+^ pump and then the maintenance of a steady state of [Ca^2+^]. When 100 μM cADPR or 10 mM caffeine was added after the steady state in the absence or presence of 10 μM tetracaine, an increase in [Ca^2+^] outside the vesicles was seen (Figures 2a and b). Tetracaine pretreatment blocked caffeine- but not cADPR-induced increase in extravesicular [Ca^2+^] (Figures 2a and b). These results support the hypothesis that cADPR-induced increase in resting [Ca^2+^]_i_ in cardiac myocytes did not involve RyRs.

### cADPR induces α-actin tyrosine dephosphorylation and its dissociation from PLB

**Figure 3:**
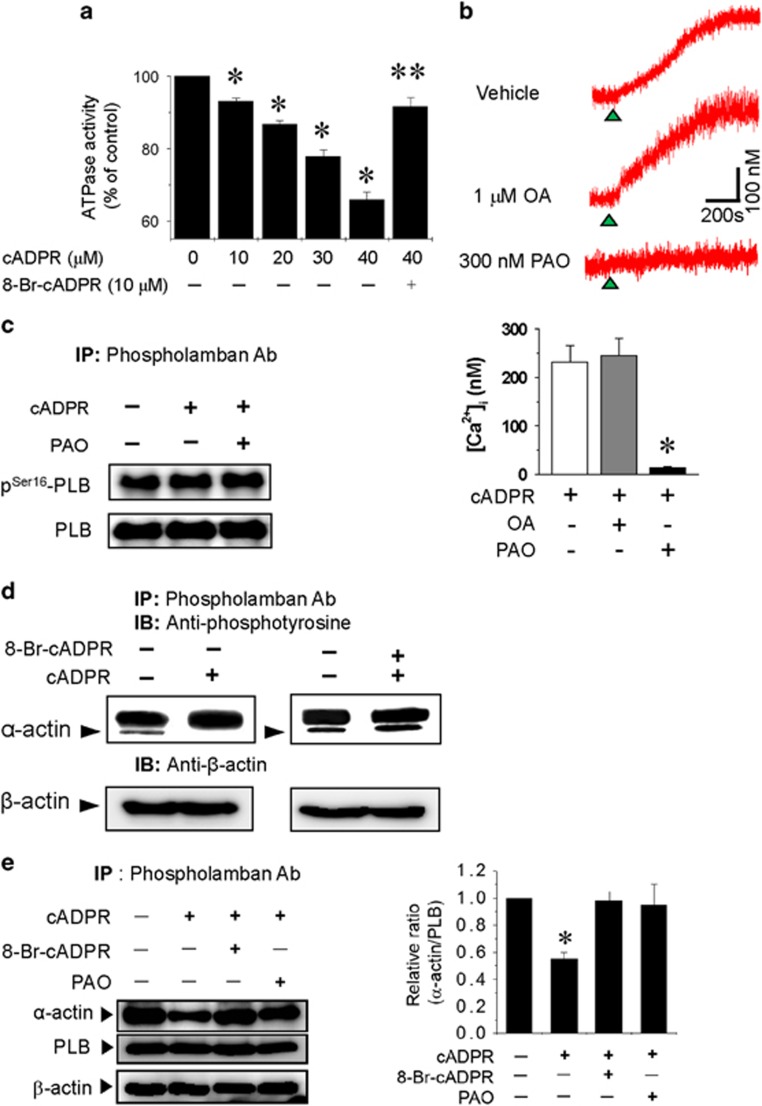
cADPR dissociates α-actin from PLB by tyrosine dephosphorylation and mediates an increase in resting [Ca2+]_i_. (**a**) Effect of cADPR on ATPase activity in rabbit SR vesicles. ATPase in intact SR vesicles was assayed as described in *Materials and Methods* in the presence of various cADPR concentrations (0, 10, 20, 30 and 40 μM) with or without a 10 min pretreatment with 8-Br-cADPR (10 μM). Columns with vertical bars denote the mean±s.e.m. of three experiments. *P<0.05 vs ATPase activity in the absence of cADPR. **P<0.05 vs ATPase activity in the presence of 40 μM cADPR without 8-Br-cADPR. (**b**) Effects of the protein phosphatase inhibitors okadaic acid (OA) and phenylarsine oxide (PAO) on cADPR-induced increase in [Ca^2+^]_i_. Fura 2-AM–loaded cells were incubated for 30 min with vehicle, 1 μM OA or 300 nM PAO before perfusion of cADPR. The tracings represent six experiments. Columns with vertical bars denote the mean±SEM of six experiments. *P<0.05 vs cADPR-induced increase in resting [Ca^2+^]_i_ in the absence of any drug. (**c**) The effect of cADPR on phosphorylation of PLB at Ser-16. Cardiac ventricular myocytes were treated with cADPR for 10 min after a 30 min pretreatment with 300 nM phenylarsine oxide (PAO). The lysates were immunoprecipitated with anti-PLB antibodies, blotted with anti-PLB antibodies or phosphor-specific antibodies against Ser-16. (**d**) The disappearance of a 42-kDa tyrosine-phosphorylated protein (arrows) after cADPR application. Cardiac myocytes were permeabilized with 0.01% saponin for 1 min and treated with 10 μM cADPR for 10 min. The lysates were immunoprecipitated with anti-PLB antibodies and blotted with anti-phosphotyrosine antibodies. β-actin served as an input control for immunoprecipitation. (**e**) The effect of cADPR on the interaction between α-actin and PLB. Cardiac myocytes were treated with 10 μM cADPR for 10 min after pretreatment with 300 nM phenylarsine oxide (PAO) for 30 min or 8-Br-cADPR for 20 min and permeabilization with 0.01% saponin for 1 min. The lysates were immunoprecipitated with anti-PLB antibodies and blotted with anti-α-actin antibodies or anti-PLB antibodies. β-actin served as an input control for immunoprecipitation. Columns with vertical bars denote the mean±s.e.m. of three experiments. *P<0.05 vs the density of protein blots from myocytes not treated with any drugs.

PLB regulates SERCA via a physical interaction.^18^ Dephosphorylated PLB inhibits SERCA activity, whereas phosphorylation of PLB by cAMP-dependent protein kinase (PKA) relieves the inhibitory effect on SERCA.^19^ PLB is regulated via phosphorylation/dephosphorylation of Ser16 and Thr17 by PKA, a Ca^2+^/calmodulin-dependent protein kinase and protein phosphatases.^16, 17, 20, 21, 22^ It has been reported that cADPR increases SERCA activity in *Xenopus* oocytes.^10, 23^ However, we found that cADPR inhibits SR ATPase activity in a concentration-dependent manner, which is completely blocked by 8-Br-cADPR pretreatment (Figure 3a). To determine whether cADPR-induced increase in resting [Ca^2+^]_i_ involves phosphorylation including PLB phosphorylation, we investigated the effect of phosphatase inhibitors. Okadaic acid (OA), a serine phosphatase inhibitor, had no effect on cADPR-induced increase in resting [Ca^2+^]_i_. In contrast, phenylarsine oxide (PAO), a tyrosine phosphatase inhibitor, blocked cADPR-induced increase in resting [Ca^2+^]_i_ (Figure 3b). However, cADPR and PAO had no effect on phosphorylation of Ser16 residue of PLB (Figure 3c). These data suggest that tyrosine phosphorylation rather than serine/threonine phosphorylation, such as that which characterizes PLB phosphorylation, is required for cADPR-mediated calcium signaling.

We identified a 42-kDa tyrosine-phosphorylated protein associated with PLB using an immunoprecipitation assay. cADPR dissociated the protein with PLB, which was blocked by 8-Br-cADPR pretreatment (Figure 3d). MALDI-TOF and ESI-Q-TOF mass spectrometry revealed that the 42-kDa protein was α-actin. cADPR decreased the association of α-actin with PLB by approximately half, which was blocked by PAO pretreatment (Figure 3e). These results indicate that cADPR activates a protein tyrosine phosphatase that dephosphorylates tyrosine residues on α-actin and dissociates α-actin from PLB.

**Figure 4:**
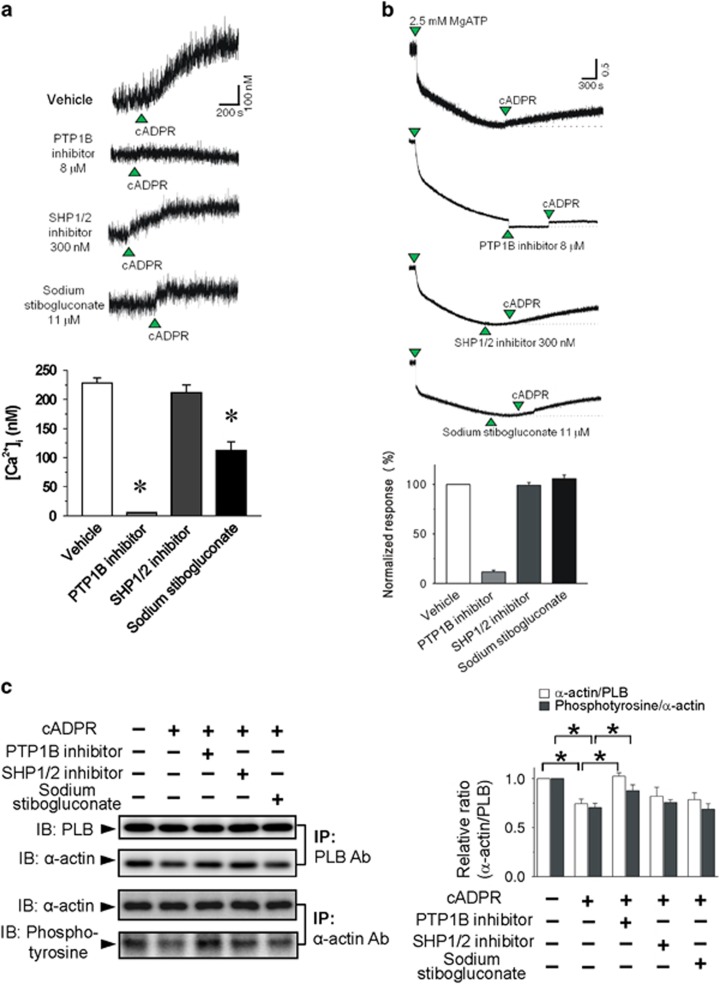
Effects of a PTP1B inhibitor, an SHP1/2 inhibitor and stibogluconate on cADPR-induced increase in resting [Ca^2+^]_i_ in isolated rabbit ventricular myocytes (**a**) and isolated SR vesicles (**b**). (**a**) Fura 2- AM loaded myocytes were incubated with 8 μM PTP 1B inhibitor, 300 nM SHP1/2 PTPase inhibitor or 11 μM sodium stibogluconate for 10 min before perfusion with cADPR. Columns with vertical bars represent a quantitative summary of the maximum [Ca^2+^]_i_ increase after cADPR addition from five experiments. *P<0.05 vs cADPR-induced [Ca^2+^]_i_ increase with vehicle pretreatment (**b**) Rabbit SR vesicles (0.5 mg per 1 ml cuvette) suspended in a cuvette were mixed with 1 μM Fura-2 lowAff. MgATP (2.5 mM) was added to the mixture to take up Ca^2+^ into the SR. After the uptake reaction achieved a steady state, 8 μM PTP 1B inhibitor, 300 nM SHP1/2 PTPase inhibitor or 11 μM sodium stibogluconate was added to the mixture before the addition of 100 μM cADPR. Columns with vertical bars represent a quantitative summary of the maximum 340/380 nm excitation ratio for Fura-2 LowAff after cADPR addition. Columns with vertical bars represent a quantitative summary of the [Ca^2+^] increase after cADPR addition from five experiments. *P<0.05 vs cADPR-induced [Ca^2+^]_i_ increase with vehicle pretreatment (**c**) The effect of cADPR on cADPR-mediated decrease in the association of α-actin with PLB and the tyrosine phosphorylation of α-actin. The SR vesicles were pretreated with 100 μM cADPR in the presence of 8 μM PTP1B inhibitor, 300 nM SHP1/2 PTPase inhibitor or 11 μM sodium stibogluconate. The SR lysates were immunoprecipitated with anti-PLB or -α-actin antibodies, run on SDS-PAGE gels, and blotted with anti-α-actin or anti-PLB or anti-phosphotyrosine antibodies. The western blot data is from eight independent sets of experiments. Columns with vertical bars denote the mean±s.e.m. of eight experiments. *P<0.05.

We further investigated the effect of other specific inhibitors of tyrosine phosphatase on cADPR-induced increase in resting [Ca^2+^]_i_ in isolated rabbit ventricular myocytes and in extravesicular [Ca^2+^] in SR vesicles (Figures 4a and b). The pharmacological inhibition of PTP1B but not SHP1/2 blocked cADPR-induced increase in resting [Ca^2+^]_i_ in ventricular myocytes and extravesicular [Ca^2+^] in SR vesicles. cADPR-mediated decrease of α-actin association with PLB was abolished by PTP1B but not by SHP1/2 inhibition (Figure 4c). These results suggest that α-actin tyrosine dephosphorylation by PTP1B is required for cADPR-induced increase in resting [Ca^2+^]_i_ in cardiac myocytes.

### α-actin integrity is required for cADPR-induced increase in resting [Ca^2+^]_i_ in isolated rabbit ventricular myocytes

**Figure 5:**
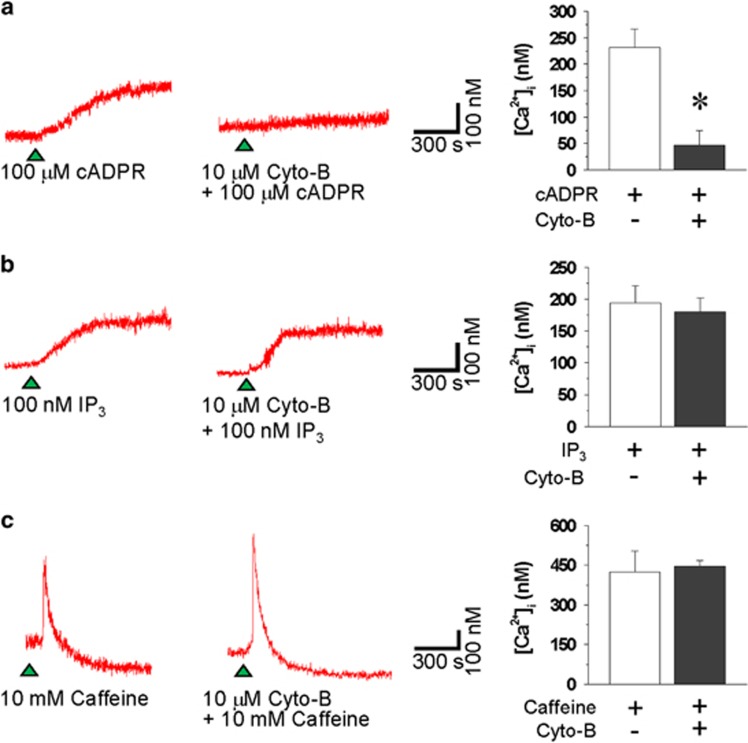
Effects of cytochalasin B on cADPR-, IP_3_- or caffeine-induced increases in resting [Ca^2+^]_i_ in isolated rabbit ventricular myocytes. cADPR (100 μM) (**a**) and IP_3_ (100 nM) (**b**) were dialyzed via a patch pipette. The arrowheads indicate the starting point of cADPR or IP_3_ perfusion. Caffeine (10 mM) (**c**) was administered to the bath. Fura 2-AM-loaded cells were incubated for 30 min with 10 μM cytochalasin B (cyto-B) before the administration of cADPR, IP_3_ or caffeine. The tracings represent six to eight experiments. Columns with vertical bars denote the mean±s.e.m. of six to eight experiments. *P<0.05 vs cADPR-induced [Ca^2+^]_i_ increase in the absence of cyto-B.

Our results clearly showed that the dissociation of α-actin from PLB plays a critical role in cADPR-induced increase in resting [Ca^2+^]_i_. To determine whether the physical interaction between α-actin and PLB was related to cADPR-induced increase in resting [Ca^2+^]_i_, we investigated the effect of cytochalasin B, a disruptor of F-actin, on cADPR-induced increase in resting [Ca^2+^]_i_. Pretreatment of the cells with cytochalasin B completely blocked cADPR-induced increase in resting [Ca^2+^]_i_ (Figure 5a), without affecting IP_3_- or caffeine-induced increase in resting [Ca^2+^]_i_ (Figures 5b and c). These results suggest that a physical interaction between α-actin and PLB is required for cADPR-induced increase in resting [Ca^2+^]_i_.

### cADPR-induced inhibition of SERCA is dependent on α-actin integrity and PTP1B activity in isolated rabbit ventricular myocytes

**Figure 6:**
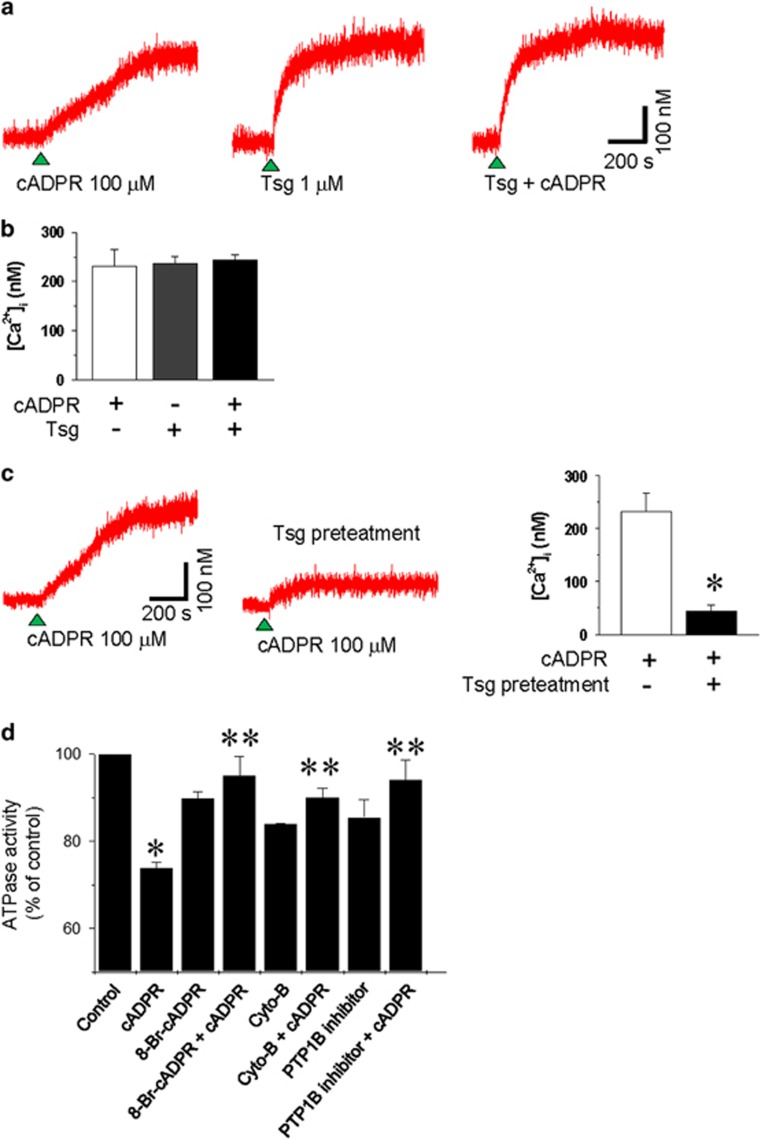
cADPR induces an increase in resting [Ca2+]_i_ by inhibiting α-actin integrity-dependent SERCA and PTP1B activity. (**a**) The effects of intracellular cADPR, thapsigargin (Tsg), and both on [Ca^2+^]_i_. cADPR (100 μM) (left), Tsg (1 μM) (middle), or both (right) were applied to the cytosol via a patch pipette. (**b**) The average values of the [Ca^2+^]_i_ increase induced by cADPR, Tsg, or both. The columns with vertical bars denote the mean±s.e.m. of six experiments. (**c**) The effect of intracellular cADPR on [Ca^2+^]_i_ in the absence or presence of 1 μM Tsg. Fura 2-AM–loaded cells were incubated for 20 min with 1 μM Tsg before cADPR perfusion. Columns with vertical bars denote the mean±s.e.m. of six experiments. *P<0.05 vs cADPR-induced [Ca^2+^]_i_ increase in the absence of Tsg. (**d**) Effect of 8-Br- cADPR, cytochalasin B, and PTP1B inhibitor on cADPR-mediated inhibition of ATPase activity. ATPase in intact SR vesicles was assayed as described in the Materials and Methods section in the presence of 40 μM cADPR with or without a 10 min pretreatment with 10 μM 8-Br-cADPR, 10 μM cytochalasin B and 8 μM PTP 1B inhibitor. Columns with vertical bars denote the mean±s.e.m. of three experiments. *P<0.05 vs the ATPase activity of control. **P<0.05 vs ATPase activity in the presence of 40 μM cADPR.

We next investigated the effect of thapsigargin, an SERCA inhibitor, on cADPR-induced increase in resting [Ca^2+^]_i_. Direct application of thapsigargin in the cytosol via a patch pipette slowly increased resting [Ca^2+^]_i_ in a manner similar to that of cADPR, although the effect of thapsigargin was much more potent and slightly more rapid than that of cADPR (Figure 6a). However, the combined application of thapsigargin and cADPR in the cytosol did not have an additive effect on increase in resting [Ca^2+^]_i_ (Figures 6a and b). Additionally, thapsigargin pretreatment blocked cADPR-induced increase in resting [Ca^2+^]_i_ (Figure 6c). cADPR inhibited SERCA activity in isolated cardiac SR vesicles in a dose-dependent manner that was blocked by 8-Br-cADPR pretreatment (Figure 3a). cADPR-mediated inhibition of SERCA activity was blocked by α-actin disruption and PTP1B inhibition (Figure 6d). These results suggest that cADPR induces an increase in resting [Ca^2+^]_i_ by SERCA inhibition via PTP1B -mediated α-actin dephosphorylation.

## Discussion

Previous studies have reported that cADPR is a specific agonist of RyR channels and induces Ca^2+^ release by sensitizing the RyR to cytosolic Ca^2+^.^24, 25, 26, 27, 28, 29, 30, 31, 32, 33^ Lukyanenko *et al.*^10^ reported that cADPR activates SERCA and potentiates a Ca^2+^ release in saponin-permeabilized rat ventricular myocytes. However, we demonstrated that cADPR inhibits SERCA in intact rabbit ventricular myocytes. This discrepancy may be due to experimental conditions such as the manner in which nucleotides such as ATP are included in the reaction. The report showed that cADPR activates SERCA by monitoring SR Ca^2+^ uptake in the presence of rheutenium red and ATP. Different from that result, we showed that cADPR inhibits SERCA under conditions closer to the actual physiological conditions in the absence of rheutenium red.

Ca^2+^ transient is a transient increase of calcium in the cell that peaks and gradually decreases during the cardiac action potential. Ca^2+^ transient is exclusively dependent on Ca^2+^ influx through L-type calcium channels. Excitation-contraction (EC) coupling in cardiac myocytes is mediated by a mechanism known as calcium-induced calcium release (CICR) where Ca^2+^ entry via L-type calcium channels induces calcium release from ryanodine-sensitive Ca^2+^ stores.^34, 35^ In cardiomyocytes, a cytosolic injection of 8-amino-cADPR reduces Ca^2+^ transient and contractions, indicating that cADPR increases EC coupling.^29^ In addition, photoreleased cADPR induces an increase in the magnitude and frequency of whole cell Ca^2+^ transient. In this study, we focused on the effect of cADPR on resting [Ca^2+^]_i_ but not Ca^2+^ transient. We investigated the effect of cADPR on resting [Ca^2+^]_i_ at higher concentrations than physiological level. We observed that relatively high concentrations of cADPR inhibit rather than activate SERCA activity, resulting in an increase in resting [Ca^2+^]_i_ in cardiac myocytes. The endogenous level of cADPR in the heart is 1.04–150 pmol mg^−1^, which corresponds to 30 nM–4.5 μM depending on experimental conditions.^36, 37^ Hypoxia increases cADPR concentration by twofold in the second-order branches of the pulmonary artery and by 10-fold in the third order branches.^38^ These results suggest that cADPR level could increase up to approximately 50 μM during specific pathological states in the heart. It has been known that myocardial ischemia, a hypoxic condition induced by blood flow restriction, increases Ca^2+^ concentration in cardiomyocytes, and it is associated with abnormal cardiac function.^13^ Cardiac ADPR cyclase, a protein distinct from CD38 or the archetypical ADPR cyclase from *A. californica*, has not been cloned to date.^39^ To verify the direct relationships between higher level of cADPR and ischemic injury, the identification of cardiac ADPR cyclase is necessary. The present study suggests that high levels of cADPR under pathophysiological conditions might decrease SR load and inhibit CICR, resulting in the inhibition of excitation-contraction (EC) coupling in cardiac myocytes.

Our results show that α-actin is involved in cADPR- but not IP3- or caffeine-induced increase of [Ca^2+^]_i_. The disruption of α-actin blocked cADPR- but not IP_3_- or caffeine-induced increases in resting [Ca^2+^]_i_, suggesting that α-actin integrity is required for cADPR action. Interestingly, α-actin was associated with PLB, and their association was decreased by cADPR treatment. The cADPR-mediated dissociation was inhibited by PTP1B inhibition. Our results suggest that cADPR activates PTP1B, which causes tyrosine dephosphorylation of α-actin.

Interestingly, although cADPR and IP_3_ act on the same calcium stores, only cADPR-mediated calcium response was sensitive to the disruption of α-actin integrity. IP_3_ mediates calcium release by direct action on IP3 receptor in SR vesicles, whereas cADPR mediates it indirectly via PTP1B, possibly resulting in the sensitivity difference to α-actin disruption.
